# An Item-Level Systematic Review of the Presentation of Attention Deficit Hyperactivity Disorder (ADHD) in Females

**DOI:** 10.1192/bjo.2024.83

**Published:** 2024-08-01

**Authors:** Annabelle Xiao Hui Lim, Tamara Williams, Louise Horstmann, Anita Thapar, Joanna Martin

**Affiliations:** 1 Cardiff University School of Medicine, Cardiff, United Kingdom; 2 Division of Psychological Medicine & Clinical Neurosciences, Cardiff, United Kingdom

## Abstract

**Aims:**

Sex differences in the prevalence of ADHD are well reported in the literature, with childhood ADHD being diagnosed 7–8 times more frequently in males than females, despite a population sex ratio of 3–4:1. A recent consensus statement argued that ADHD is under-identified and under-diagnosed in the UK, and this is especially concerning with regards to females. This systematic review aims to investigate specific symptoms characterising the manifestation of ADHD in females compared with both males with ADHD and females without ADHD.

**Methods:**

A systematic search of eligible studies was conducted using predefined search criteria across six databases (Ovid MEDLINE, Ovid EMBASE, Ovid APA PsycINFO, ProQuest, EBSCO ERIC and EBSCO British Education Index), in line with a registration protocol on PROSPERO. Eligible studies included those with statistical analysis comparing ADHD, impact or co-occurring mental health difficulties at the item level, which compared ADHD symptoms in both sexes, or contrasted females with and without ADHD. Studies that exclusively reported total scores without item-level statistical results were excluded. A total of 5,378 articles were identified in the search and 13 studies met the criteria for inclusion.

**Results:**

Outcomes from 13 studies were analysed thematically. 7 studies looked at ADHD at an item level, while 7 studies explored disparities in impairment or other items. Of the eligible studies, 12 compared males and females with ADHD and 4 compared females with and without ADHD. 7 studies focussed on children with ADHD and 6 on adults. Preliminary results from 3 studies of ADHD symptoms in children indicated sex differences in hyperactive and impulsive symptoms: males were more likely to exhibit symptoms such as fidgeting and difficulty remain seated, while females exhibited higher rates of excessive talking and interrupting. Sex differences in impairment showed mixed results. Females with ADHD endorsed self-reported items related to mind-wandering and parent-reported impairment, including friendship difficulties, more than females without ADHD. Overall, the analysis of the results suggested that most studies do show some sex differences in ADHD and impairment items.

**Conclusion:**

While current studies of individuals diagnosed with ADHD highlight important sex differences, the limited number of direct investigations and predominant focus on total symptoms underscore the need for further research. Item-level analysis of symptoms and their impact is essential in exploring how sex influences the associations between ADHD, risk factors and functional outcomes. Recognising potential sex differences is essential for improving ADHD assessment in females and later life outcomes.

